# Premature Activation of Immune Transcription Programs in Autoimmune-Predisposed Mouse Embryonic Stem Cells and Blastocysts

**DOI:** 10.3390/ijms21165743

**Published:** 2020-08-11

**Authors:** Oktay Kirak, Eugene Ke, Kevin Y. Yang, Anna Schwarz, Lars Plate, Amy Nham, Justin R. Abadejos, Anna Valencia, R. Luke Wiseman, Kathy O. Lui, Manching Ku

**Affiliations:** 1 Department of Immunology and Microbial Science, The Scripps Research Institute, 10550 North Torrey Pines Road, La Jolla, CA 92037, USA; aschwarz@scripps.edu (A.S.); nhamamy@gmail.com (A.N.); Jabadejo@scripps.edu (J.R.A.); avalencia@scripps.edu (A.V.); 2 Division of Pediatric Hematology and Oncology, Department of Pediatrics and Adolescent Medicine, Faculty of Medicine, Medical Center—University of Freiburg, Mathildenstraße 1, 79106 Freiburg, Germany; 3 Salk Institute for Biological Studies, 10010 North Torrey Pines Road, La Jolla, CA 92037, USA; ek7kn@virginia.edu; 4 Department of Chemical Pathology and Li Ka Shing Institute of Health Sciences, Prince of Wales Hospital, The Chinese University of Hong Kong, Hong Kong, China; styangyi@live.cn (K.Y.Y.); kathyolui@cuhk.edu.hk (K.O.L.); 5 Department of Molecular Medicine, The Scripps Research Institute, 10550 North Torrey Pines Road, La Jolla, CA 92037, USA; lars.plate@vanderbilt.edu (L.P.); wiseman@scripps.edu (R.L.W.)

**Keywords:** embryonic stem cells, autoimmunity, non-obese diabetic mice, predisposition, RNA-Seq, ChIP-Seq, cytokines, chemokines, multi-omic analyses

## Abstract

Autoimmune diabetes is a complex multifactorial disease with genetic and environmental factors playing pivotal roles. While many genes associated with the risk of diabetes have been identified to date, the mechanisms by which external triggers contribute to the genetic predisposition remain unclear. Here, we derived embryonic stem (ES) cell lines from diabetes-prone non-obese diabetic (NOD) and healthy C57BL/6 (B6) mice. While overall pluripotency markers were indistinguishable between newly derived NOD and B6 ES cells, we discovered several differentially expressed genes that normally are not expressed in ES cells. Several genes that reside in previously identified insulin-dependent diabetics (Idd) genomic regions were up-regulated in NOD ES cells. Gene set enrichment analysis showed that different groups of genes associated with immune functions are differentially expressed in NOD. Transcriptomic analysis of NOD blastocysts validated several differentially overexpressed Idd genes compared to B6. Genome-wide mapping of active histone modifications using ChIP-Seq supports active expression as the promoters and enhancers of activated genes are also marked by active histone modifications. We have also found that NOD ES cells secrete more inflammatory cytokines. Our data suggest that the known genetic predisposition of NOD to autoimmune diabetes leads to epigenetic instability of several Idd regions.

## 1. Introduction

Autoimmune diabetes, also known as type-1 diabetes (T1D), is a multifactorial disease with a well-known and growing number of disease-relevant genes [1]. In addition to human leukocyte antigen (HLA) class II alleles, many other non-HLA genes have been identified, including INS, PTPN22, CTLA4, IL2RA and IFIH1, with more to come [2,3,4,5,6]. 

In addition to the genetic makeup, environmental factors are known to contribute to disease onset. However, such triggers are composed of a highly heterogeneous group of factors that have been implicated to promote disease onset [7]. These include infections (e.g., enteroviruses) [8,9], birth (e.g., birth weight, or caesarean section) [10,11] and dietary factors (e.g., gluten) [12,13,14], as well as β-cell stress (e.g., overweight, puberty or trauma) [12]. Further, increased secretion of cortisol caused by psychological stress has also been postulated to play a role in the onset of T1D [15]. Recently, we showed that a B cell Receptor (BCR) mouse model derived from an early pancreas-infiltrating B cell from NOD mice expressed several nucleic acid sensing receptors [16]. However, no single environmental trigger can induce the onset of T1D. It is more likely that certain environmental triggers combined with genetic risk factors increase the potential to develop T1D.

The non-obese diabetic (NOD) mouse model spontaneously develops T1D and shares many similarities with the pathologies found in human patients [17,18]. The NOD strain was initially generated in Japan by selecting for a cataract-prone strain from the outbred ICR line [19,20]. At generation six of the ICR inbreeding program, Ohtori and colleagues started to establish two additional separate inbred lines based on high or normal fasting serum glucose levels. As the breeding program continued, mice emerged with glucosuria and hyperglycemia in the initially normoglyemic substrain, which became the NOD strain. Additionally, a normoglycemic substrain was established from the initial hyperglycemic substrain, which became the NON strain [21]. Although the cataract trait was present initially during the breeding programs, it was lost later in both NOD and NON strains. Contrary to NOD, the inbred C57BL/6 (herein referred to as B6) strain develops no autoimmune diseases and has low susceptibility to tumors [22]. It is the most commonly used mouse strain in a broad range of research areas.

Here, we report on the derivation and characterization of embryonic stem cells from the two inbred strains NOD and B6. While the expression of pluripotency genes was similar among NOD and B6 strains and at comparable levels to existing V6.5 (B6x129/JaeF1) ES cells, a detailed transcriptional assessment between the two inbred strains revealed several significant differences, such as the overexpression of γ-Crystalline D and several known insulin-dependent diabetes (Idd) and additional immune-related genes. Strikingly, analysis of NOD and B6 blastocysts confirmed early expression of some Idd genes that we had initially identified in ES cells, validating our observations of premature activation of the immune transcriptional program in the genetic predisposed NOD strain.

## 2. Results

### 2.1. Derivation of ES Cells from the Inbred Strains NOD and B6

ES cells can be derived from the inner cell mass of pre-implantation blastocysts. Two compounds, a MEK inhibitor and a GSK3 inhibitor, have been identified that facilitate derivation of ES cells from un-manipulated as well as cloned (a.k.a. somatic cell nuclear transfer) NOD blastocysts [16,23,24,25]. The culture conditions are referred to as 2i. We successfully derived 22 NOD ES cell lines from 24 fertilized NOD blastocysts (over 90% efficiency). Given that only male ES cell lines are used for gene targeting, we focused our further characterization on male ES cell lines (NOD-5 and -6). We also derived ES cell lines from other mouse models as controls: NZB/BlNJ (NZB), which is predisposed to several autoimmune diseases, such as hemolytic anemia that closely resembles symptoms of systematic lupus erythematosus; and BL/6 (B6), a pure background mouse strain that does not develop diabetes. Additionally, we used V6.5 (C57B6 x 129S4/SvJae), a mixed background ES cell line that has been extensively and successfully used for gene targeting purposes [26]. Alkaline phosphatase staining, and embryoid body formation revealed no obvious differences among the tested ES cell lines (Figure S1a,b). Quantitative real-time PCR (RT-PCR) was performed to determine the expression levels of pluripotency genes Pou5f1 (Oct4), Sox2 and Nanog, as well as markers indicating differentiation, such as Brachyury, Bmp2, Bmp4, Nestin, Gata4, Sox17 and Hoxa7a. Overall, the expression levels of the pluripotency genes showed little to no difference, while none of the tested ES cell lines expressed markers indicative of differentiation (Figure S1c). In addition, immunofluorescence experiments confirmed the expression of pluripotency genes Oct3/4 and Sox2 at protein levels (Figure S1d). As shown in Figure S1e, germline transmission was achieved in both B6 and NOD ES lines.

### 2.2. Transcriptional and Proteomic Comparison between NOD and B6 ES Cells

**Figure 1:**
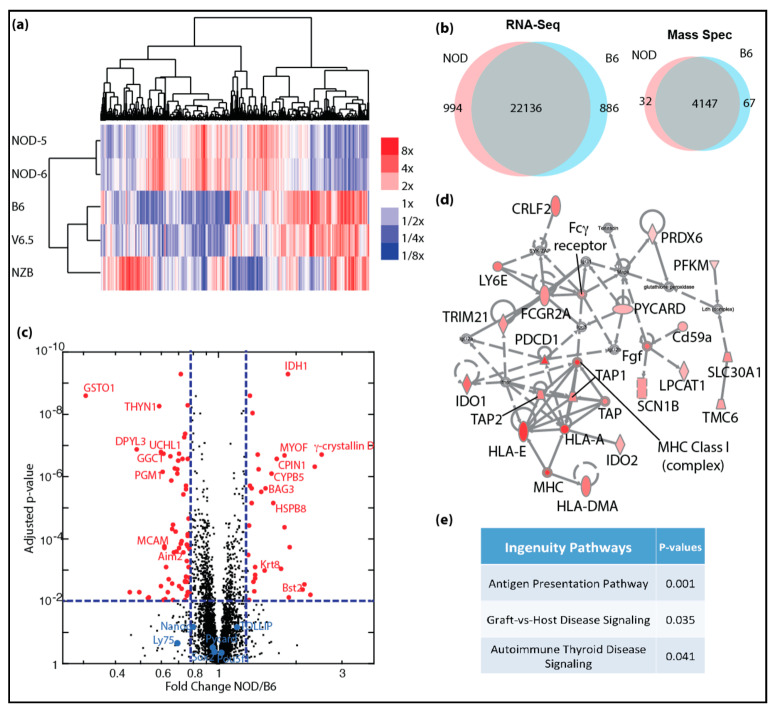
Genome-wide transcriptomic and proteomic analyses of autoimmune-prone ES cell lines: (**a**) heatmap shows clustering of differentially expressing genes identified in mRNA-Seq for NOD, B6, V6.5 and NZB ES cell lines, color scale bar shows expression level relative to average; (**b**) venn diagrams show overlap of genes with similar expression levels (left) and protein expression (right) in NOD and B6 ES cells; (**c**) volcano plot shows significantly increased protein expression in NOD (red dots, right) and in B6 (red dots, left) ES cells. (**d**) Ingenuity pathway analysis shows majority of proteins in the inflammation pathway are up-regulated (red) with 2-fold or more up-regulation and FDR < 0.05. (**e**) Ingenuity canonical pathways that are enriched in genes that are up-regulated in NOD ES cells.

In order to study if early embryonic gene expression plays a role in predisposition in autoimmune diabetes, we performed stranded mRNA-Seq on ES cell lines from NOD, B6, NZB and V6.5 (Figure 1a). All ES cells, including V6.5 were cultured under the same 2i conditions in the absence of feeder cells to minimize variability. For primary analysis, we compared the results with RNA-Seq data derived from several mouse tissues available from the ENCODE consortium [27] (Figure S2). Hierarchical clustering of gene expression data demonstrated that NOD, B6 and NZB ES cell lines derived from our lab cluster closely together with published expression data from V6.5, further confirming their similarity to existing embryonic stem cell lines and not to differentiated tissues (Figure S2). Interestingly, the B6 ES cell line “bruce4” showed a transcriptionally distinct profile from all the ES cell lines analyzed, which may partly explain its limited success for gene targeting approaches, meaning that germline transmission was often achieved, but was not reliable [28]. Nonetheless, our RNA-seq data demonstrated that bruce4 has a similar but yet distinguishable profile to all other ES cell lines including V6.5.

Given that NOD and B6 ES cell lines were similarly pluripotent, we closely investigated whether there are differentially expressed genes that would give clues to the potential to develop autoimmune diabetes. We focused our analysis on NOD ES cells and compared them to B6. As shown in Figure 1a, NOD ES lines clustered closely together, while B6 clustered together with V6.5. Interestingly, we found a cluster of genes that was uniquely expressed in NOD ES cells, while another cluster was unique to B6. In total, 994 genes were uniquely overexpressed in diabetes-prone NOD ES cell lines, while 886 genes were overexpressed in B6 ES cell lines (Figure 1b). To our surprise, among the top genes that were differentially expressed in ES cells and most abundant in NOD was γ-Crystallin D. γ-Crystallin D is normally expressed specifically and exclusively in the lens of the eye [5,6]. Unlike α-Crystallins, which are more ubiquitously expressed and can function as heat-shock proteins, γ-Crystallins have no heat-shock protein function, are very restricted in their expression and overexpression has been linked to cataract development [29,30].

In order to validate and quantify the differences in genes products and to validate that overexpressed transcripts are translated into more proteins, we performed quantitative proteomics using tandem mass tags (TMT) [9,10,11]. We were able to detect a total of 4147 proteins using TMT. Among these proteins, 99 proteins were differentially expressed in NOD (32 up-regulated) and B6 (67 up-regulated) ES cells including γ-Crystallin D in NOD and Aim2 in B6 (right panel in Figure 1b; red dots in Figure 1c). We were also able to demonstrate that pluripotency genes, such as Oct4 (Pou5f1), Nanog and Sox2 are equally expressed at protein levels in NOD and B6 ES cell lines (blue dots in Figure 1c). We performed gene set enrichment and pathway analysis using the Ingenuity Pathway Analysis algorithm (IPA, Qiagen), and found that genes that are up-regulated in NOD ES cells are preferentially enriched in pathways related to inflammation and immune functions (Figure 1d,e).

### 2.3. De-repression of Idd Genes and Other Immune-Related Genes in Diabetic-Prone NOD ES Cells

**Figure 2:**
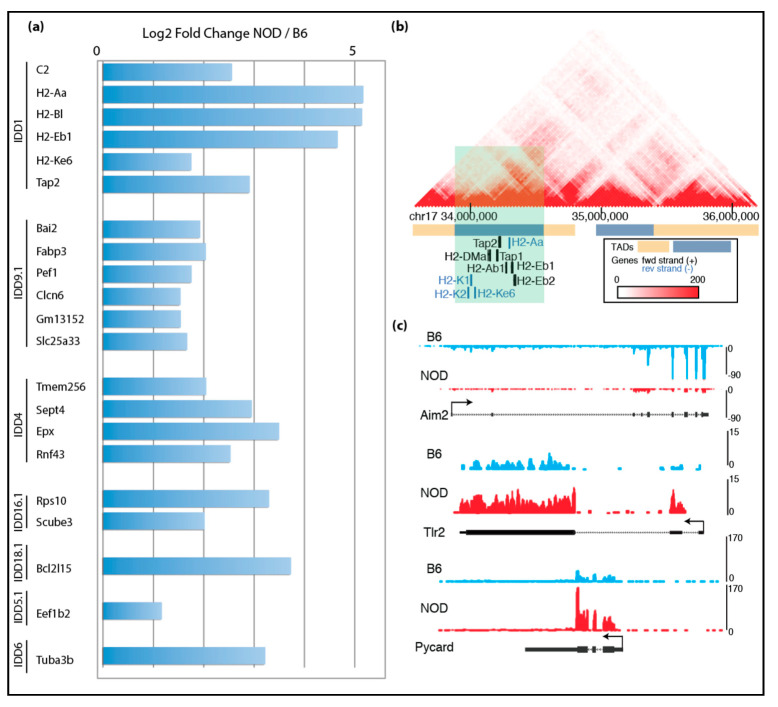
Transcriptomic comparison of NOD and B6 ES cells: (**a**) mRNA-Seq data show up-regulated genes found in Idd regions of NOD ES cells compared to B6 ES cells; (**b**) genes that are up-regulated in NOD Idd1 region belong to the same topological associating domain (TAD); (**c**) UCSC genome browser tracks show that Aim2 is up-regulated in B6 ES cells, while Tlr2 and Pycard are up-regulated in NOD ES cells.

Given the unexpected finding of γ-Crystallin D overexpression in NOD ES cell lines, we reasoned that other disease-relevant genes might be also activated in diabetes-prone NOD ES cells specifically, but not in systematic lupus erythematosus-prone NZB, nor in diabetes-resistant B6 and v6.5 ES cells. Thus, subsequent RNA-Seq analysis was focused on the differences among the diabetes-prone NOD ES cell lines and the diabetes-resistant B6 ES cell lines, which are both pure backgrounds. Our analysis showed premature expression of immune-related genes in insulin-dependent diabetes (Idd) regions in NOD. We cross-referenced our list of genes that were up-regulated in NOD ES cell lines with the known Idd loci and identified 58 genes within various Idds (Figure 2a). Among these genes were well-known important players of T1D, such as H2-K, H2-D, H2-L, Tap1 and Tap2. Tap1 and Tap2 are transporters for cytoplasmic peptides, are part of the MHC-I-peptide loading complex and are thus involved in antigen processing and presentation [7]. Using published Hi-C data in mouse embryonic stem cells [31], we found that Idd1, Idd4.2Q, Idd9.1 and Idd16.1 genes that are up-regulated in NOD compared to B6 localized in the same topologically associating domains (TADs), suggesting that they are transcriptionally co-regulated due to their proximity to each other and physical interaction at the genomic level (Figure 2b and Supplemental Figure S3a–d) [32].

Given that T1D is a multifactorial autoimmune disease, we further focused our analysis on immune-related genes. Strikingly, we found many other immune-related genes are differentially expressed in NOD and B6 ES cells: Aim2 (Absent in melanoma 2) was overexpressed in B6, while Tlr2 was overexpressed in NOD (Figure 2c) [8]. Aim2 plays an important role in innate immunity by functioning as a cytoplasmic DNA sensor, and by triggering activation of the inflammasome through Asc (apoptosis-associated Speck-like protein containing a caspase activation and recruitment domain). Asc is encoded by Pycard and plays a central role in recruiting procaspase-1 to the complex, which then can lead to activation of caspase-1 and the inflammasome. Interestingly, Pycard was expressed in both NOD and B6 ES cells, albeit at higher levels in NOD ES cells (Figure 2c).

### 2.4. Characterization of Diabetes-Prone and Diabetes-Resistant Preimplantation Embryos

**Figure 3:**
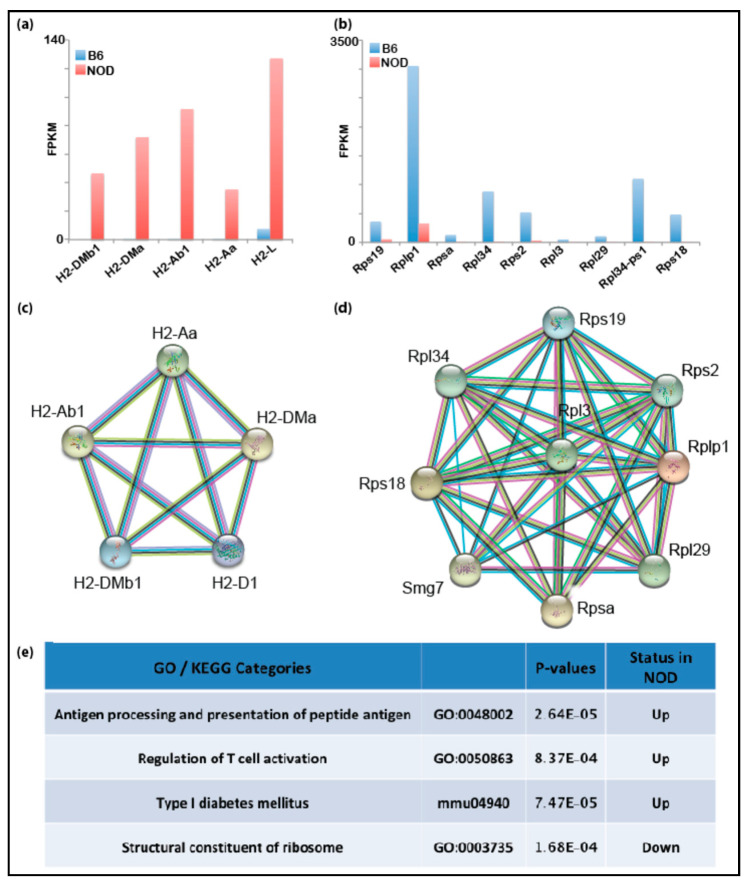
Transcriptomic analyses of NOD and pre-implantation B6 blastocysts: bar graphs show that genes in the Idd1 region that are (**a**) up-regulated in NOD blastocysts or (**b**) in B6 blastocysts. Functional protein association network analysis by STRING shows Idd regions that interact in (**c**) NOD blastocysts and (**d**) B6 blastocysts. (**e**) Gene set enrichment analysis shows that immune-related genes are up-regulated in NOD blastocysts.

Using RNA-Seq and mass spectrometry (MS) analyses, we had identified several immune-related genes that were prematurely and differentially overexpressed in diabetes-prone NOD ES cells compared to B6 ES cells. Thus, we hypothesized that genetic predisposition to T1D may manifest transcriptionally at the early embryonic stage resulting in differentially expressed immune-related genes in healthy and disease-prone embryos. Even though primary ES cells serve as a good proxy for studying transcription and chromatin states of the pre-implantation embryonic stage, it is possible that the transcription programs in NOD and B6 ES cell lines may differ from the developing embryos due to in vitro culturing conditions or stress applied to the inner cell mass (ICM) during the process of ES cell derivation. In order to validate our findings in ES cells in vivo, we assessed gene expression directly in pre-implantation embryos. We isolated the ICM from NOD and B6 blastocysts using standard immunosurgery and performed low-input RNA-Seq on ICMs isolated blastocysts using the SMART-Seq V4 Ultra-low RNA-Seq library protocol (Takarabio). Differential gene expression from NOD blastocysts showed that Idd1 and immune-related genes are also up-regulated in NOD pre-implantation blastocysts compared to B6 (Figure 3a), consistent with our gene expression data obtained from ES cells. On the contrary, genes relevant to structural constituents of ribosomes are down-regulated in NOD pre-implantation embryos compared to B6 (Figure 3b). The down-regulation of ribosomal genes is consistent with previous observation of decreased ribosome synthesis in diabetic animal models [33]. Functional protein association networks analysis by STRING showed the genes which were up- or down-regulated and formed gene networks that are functionally closely related (Figure 3c,d). We performed gene enrichment analysis and found that genes that are up-regulated in NOD blastocysts were enriched in categories for antigen processing, antigen presentation, T cell activation and Type I diabetes mellitus, while genes involved in the structural constituent of ribosomes were down-regulated (Figure 3e).

### 2.5. Epigenomic Characterization of Diabetic-Prone NOD ES Cells

**Figure 4:**
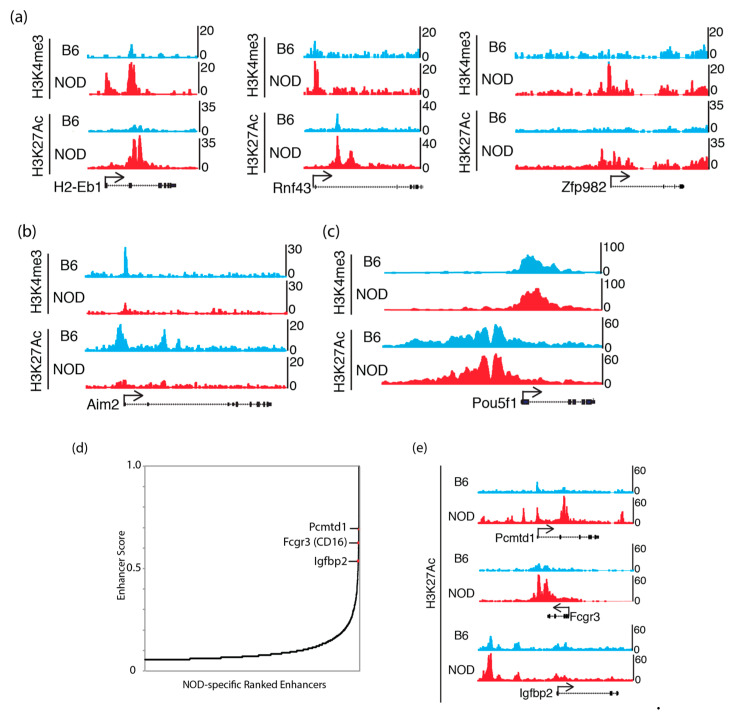
Active chromatin states in NOD and B6 ES cells: UCSC genome browser tracks show H3K4me3 and H3K27ac ChIP-Seq levels that are (**a**) increased at Idd genes: H2-Eb1, Rnf43 and Zfp982 in NOD ES cells; (**b**) increased at Aim2 in B6 ES cells and (**c**) equally enriched at the Pou5f1 (Oct4) promoter in both B6 and NOD ES cells. (**d**) NOD-specific super enhancer analysis using H3K27ac ChIP-Seq signals discovered genes that are implicated in diabetes GWAS studies. (**e**) UCSC genome browser tracks show stronger H3K27ac ChIP-Seq signals at NOD-specific super enhancers compared to B6.

To understand the potential epigenetic mechanism that contributes to the differential gene expression in NOD compared to B6 ES cells, we characterized chromatin states in ES cells, by chromatin-immunoprecipitation followed by sequencing (ChIP-Seq) to identify the genome-wide distribution of active histone mark, histone H3 trimethyl-K4 (H3K4me3), at promoter sites and active histone mark, histone H3 acetyl-K27 (H3K27ac), at enhancer sites [34,35]. We found an increase in H3K4me3 and H3K27ac in genes that were up-regulated in NOD ES cells (Figure 4a). Genes that were up-regulated in B6 ES cells, such as Aim2, also show a corresponding increase in H3K4me3 and H3K27ac (Figure 4b), while actively transcribing pluripotency factors such as Pou5f1 (Oct4) showed similar levels of these active histone modifications (Figure 4c). However, we also found a subset of these genes to show no dramatic changes in these active histone marks, suggesting a different mechanism of transcriptional de-repression or activation (Supplemental Figure S4). We also performed NOD-specific super enhancer analysis using H3K27ac ChIP-Seq signals in NOD ES cells [36] and found a subset of top super enhancers, Pcmtd1, CD16 and Igfbp2, which were previously implicated in diabetes genome-wide association studies (GWAS) [37,38] (Figure 4d). We observed an increase in H3K27ac marks in Pcmtd1, Fcgr3 and Igfbp2 (Figure 4e). This suggests that genes that may be involved in diabetes are marked by active enhancer marks.

### 2.6. NOD ES Cells Secrete Inflammatory Cytokines and Chemokines

**Figure 5:**
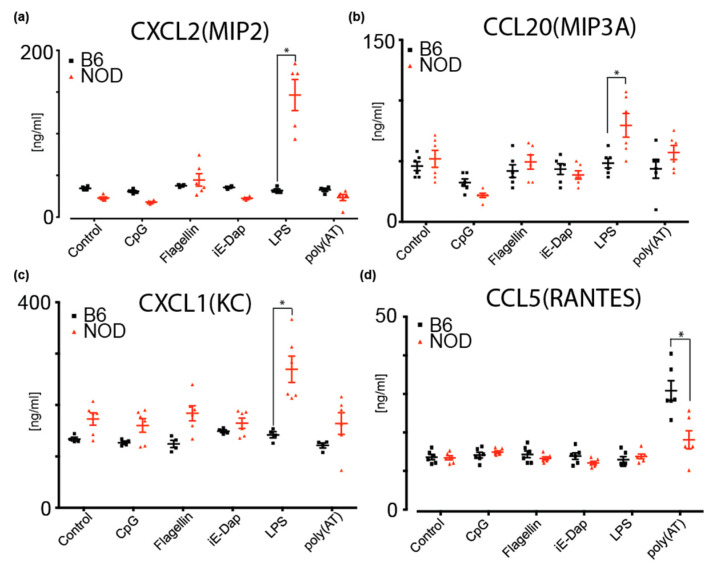
Multiplex ELISA measurements of cytokines and chemokines secreted by B6 or NOD ES cells upon stimulation by various triggers; (**a**) MIP2(CXCL2), (**b**) MIP3A(CCL20) and (**c**) KC(CXCL1) were shown to increase secretion from NOD ES cells upon LPS stimulation compared to B6 ES cells. (**d**) RANTES(CCL5) was shown to secrete more in B6 ES cells compared to NOD ES cells upon poly(AT) stimulation. * *p* < 0.001. Significance was calculated by unpaired t test, *n* = 6.

To investigate potential cytokine and chemokine secretion by NOD ES cells, we performed a multiplexed antibody-based immunoassay using Luminex xMAP mouse panels (Biorad) on cultured medium from NOD and B6 ES cells. These cytokines are implicated in inflammation and the regulation of many immune cell types and immune responses [39]. Given that we had identified several differentially expressed pattern recognition receptor (PRR) genes that are implicated in innate immune responses, we determined whether diabetes-prone NOD ES cells respond differently to inflammatory triggers compared to B6. To do so, we determined secreted cytokines upon treatment of NOD and B6 ES cells with CpG (recognized by Toll-like receptor 9, TLR9), Flagellin (recognized by TLR5), iE-DAP (recognized by Nucleotide-binding oligomerization domain-containing protein 1, NOD1), LPS (recognized by TLR4) and poly(AT), which can bind to cGAS (Cyclic GMP-AMP synthase), AIM2 (absent in melanoma 2), DAI (DNA-dependent activator of IFN-regulatory factors, also known as Z-DNA-binding protein 1, ZBP1), DDX41 (DEAD-Box Helicase 41), IFI16 (Interferon Gamma Inducible Protein 16) and LRRFIP1 (LRR Binding FLII Interacting Protein 1) (Figure 5 and Figure S5b). We found that MIP2 (CXCL2) and MIP3A(CCL20) are specifically and significantly secreted more by NOD ES cells upon LPS stimulation when compared to B6 (Figure 5a,b). As shown in Figure 5c, KC(CXCL1) baseline secretion was higher in NOD ES cells, compared to B6 ES cells but not significantly. However, upon stimulation with LPS and Flagellin, a significant increase in secretion was observed compared to B6 ES cells and baseline NOD secretion (Figure 5c). Strikingly, B6 ES cells did not respond to stimulation with LPS or Flagellin. Instead, B6 ES cells responded with a robust secretion of RANTES (CCL5) after sensing poly(AT) (Figure 5d).

## 3. Discussion

Embryonic stem cells are a critical tool to study development, pluripotency, differentiation and epigenetic reprogramming. ES cells and iPS (induced pluripotent stem) cells hold great promise to generate patient-specific cells and eventually organs to treat various diseases, including degenerative and possibly autoimmune diseases.

Here, we report on the derivation and characterization of novel mouse ES cells from diabetes-prone NOD and diabetes-resistant B6 strains. Strikingly, one of the most differentially overexpressed genes in NOD ES cells was γ-Crystallin D (Figure 1). NOD is a sister line of the cataract-prone Shionogi (CTS) line. Among the various clinical forms of inherited cataract in humans, several have been linked to mutations and aberrant expression of γ-Crystallin D [29,30]. We hypothesize that one of the factors pre-disposing CTS to develop cataract might be dysregulation of γ-Crystallin D, and that NOD has inherited this epigenetic trait.

As mentioned above, an extensive breeding program in which diabetes-prone NOD mice were crossed with various diabetes-resistant strains (e.g., B6 and B10) was implemented to identify genomic regions which cause diabetic phenotypes when present in the aforementioned healthy strains. These studies led to the identification of Idd loci that are likely to play important roles in mediating the genetic predisposition to T1D in NOD mice. A closer look at the differentially expressed genes in NOD ES cells revealed 58 genes located within the known Idd loci (Figure 2). Specifically, we found that some of the immune-related genes involved in adaptive and innate immunity are differentially expressed in diabetes-prone NOD and diabetes-resistant B6 ES cells are grouped into one of the TADs within Idd/MHC region. We analyzed chromosomal interaction in the Idd1/MHC region using published HiC data in mouse ES cells [31,32] and found that there are eight topologically associated domains (TADs) (Figure 2b and Figure S3a). Idd genes that are up-regulated in NOD ES cells were found to be located in the same TADs within the larger Idd regions. The TADs identified in the Idd4.2Q, Idd9.1 and Idd16.1 regions are displayed in Figure S3b–d, with genes that are activated in NOD ES cells highlighted. Tlr2 and Pycard are up-regulated in NOD ES cells, which is consistent with previous reports on their involvement in inflammation and diabetes [40,41,42].

On the contrary, Aim2 was found to be down-regulated in NOD compared to B6 ES cells, and was previously shown to be protective for B6 mice from developing diabetes in the presence of Steptozotocin [43], supporting the gene expression profiles predisposing to diabetes in NOD mice. Interestingly, diabetes-resistant B6 ES cells differentially overexpressed other immune-related genes, while a different set of immune-related genes was expressed at equal levels in both ES cells. This suggests that both a diabetes-prone and a diabetes-resistant epigenetic trait might be inherited.

In order to analyze differentially expressed genes in early pre-implantation, we performed RNA-Seq in blastocysts from both the diabetes-prone NOD and the diabetes-resistant strain B6 (Figure 3). Strikingly, we were able to show that diabetes-prone pre-implantation embryos from NOD mice differentially overexpressed some of the previously identified immune-related genes, such as Ifngr1 and Il22ra2. Even more fascinating was the identification of Idd-genes, such as MHC-II. As stated above, MHC-II is the gene with the strongest diabetes association. Based on our STRING analysis, we were able to identify a cluster of genes involved in, e.g., antigen processing and presentation as well as protein folding.

We hypothesize that the premature activation of the immune-related genes in NOD ES cells may also be accompanied by the modulation of the chromatin states. We performed H3K4me3 and H3K27ac ChIP-Seq experiments to identify gene promoters and enhancers marked by these active histone modifications. We found that a subset of activated genes in NOD ES cells is also marked by increased H3K4me3 and H3K27ac ChIP-Seq signals as compared to B6 ES cells. Interestingly, increased H3K4me3 and H3K27ac in up-regulated genes in NOD ES cells correlate to promoters with lower CpG content (Figure 4a, Figure S4). Using H3K27ac ChIP-Seq signals, we also identify super enhancers specific in NOD ES cells. When we examined the super enhancers in NOD ES cells, we found that Pcmtd1, Fcgr3 and Igfbp2 are implicated in human diabetes from GWAS studies. Moreover, NOD mice with deficient Fcgr3 exert a protective effect against diabetes, suggesting Fcgr3′s role in the pathogenesis of diabetes in NOD mice [44]. Circulating Igfbp2 protein is up-regulated in type-1 diabetic patients, suggesting increased risks [45,46]. Together, NOD ES cells display modified chromatin and transcriptional states favoring the activation of immune-related genes.

Among the genes that we identified to be differentially expressed in NOD and B6 ES cells, many were known to be involved in innate and adaptive immune responses. While some cytokines promote a tolerogenic environment, such as TGF-b or IL-10, others are known to support a diabetogenic environment, such as TNF and IL-6 [47]. Thus, we investigated whether NOD and B6 ES cells are already capable of responding to certain triggers of the innate immune system, and consequently might secret cytokines. Strikingly, in addition to gene expression and chromatin states, we also found that NOD and B6 ES cells secreted different inflammatory cytokines and chemokines upon stimulation of PRRs. In the presence of LPS, which mimics bacterial infections, NOD ES cells were induced to secret high levels of MIP2, MIP3A and KC contrary to B6 ES cells. The induction of MIP2 secretion in NOD ES cells is consistent with a previous report on MIP2 induction upon LPS injection in a chemically induced diabetic mouse model [48]. Moreover, it was previously observed that the inflammatory effect of bacterial infection is prolonged in the db/db mouse model, which might be explained, in part, by the stronger induction of MIP2 by LPS in our experiments [49]. Additionally, an increase in KC secretion by NOD ES cells was also observed upon stimulation with Flagellin. [39,48,49,50,51]

## 4. Materials and Methods

### 4.1. Mice, Preimplantation Embryos and ES Cell Derivation

All animal experiments were approved by the Scripps Research Institute Institutional Animal Care and Use Committee. Embryonic stem cells were derived from blastocysts isolated from C57BL/6, NOD and NZB mice and cultured in the presence of 2i inhibitors for MEK and GSK3 (PD032590 and CHIR99021), as described previously [16,23,24]. For germline transmission test, B6 ES cells were injected into albino blastocysts. Resulting chimeric mice were crossed with albino mice. Non-albino offspring indicated germline transmission of B6 ES cells. B NOD ES cells were injected in non-albino blastocysts. Resulting chimeric mice were bred with albino mice. Albino offspring indicated germline transmission of NOD ES cells.

### 4.2. Alkaline Phosphatase Detection

Alkaline phosphatase staining was performed according to the manufacturer’s instructions (SigmaAldrich, St. Louis, MO, USA).

### 4.3. Embryoid Body Formation

NOD or B6 ES cells were cultured in 2i conditions until about 80–90% confluent. ES cells were then trypsinized and plated at 50 to 60 thousand cells per cm^2^ on low-binding 6-well culture plates, with shaking overnight at room temperature at 95 rpm in ES culture media without 2i.

### 4.4. Quantitative Real-Time PCR

RNA was isolated from NOD or B6 ES cells using the NucleoSpin RNA mini kit for RNA purification (Macherey-Nagel, Düren, Germany). RNA was converted into cDNA using qScript cDNA supermix (Quanta Bioscience, Beverly, MA, USA), 5% of 20ul reaction was used per primer set in quantitative real-time PCR (RT-PCR) using a (Bio-rad, Hercules, CA, USA) real-time PCR instrument. Pluripotency gene primers were previously established [52].

### 4.5. Immunofluorescence

B6 and NOD ES cells were cultured on chamber slides and then fixed with 4% paraformaldehyde for 30 min at room temperature. Fixed cells were then permeabilized with 0.2% Triton-X100 and blocked with goat serum for 2 h at 4 °C. Permeabilized cells were then incubated with primary antibodies (Oct3/4 and Sox2 from Abcam, Cambridge, UK) overnight at 4 °C then incubated with secondary antibodies with Alexa Fluor (ThermoFisher, Waltham, MA, USA) for 1hr prior to imaging on a fluorescence microscope.

### 4.6. Stranded mRNA-Seq

RNA was isolated from B6 and NOD mouse ES cells using Nucleospin RNA kit and treated with DNase according to the manufacturer’s protocol (Machery-Nagel). mRNA-Seq library was prepared using TruSeq stranded mRNA sample prep kit (Illumina, San Diego, CA, USA). Between 17 and 21 million uniquely alignable reads were obtained from each RNA-Seq library. RNA-Seq reads were aligned using STAR (v2.3.0e). Gene expression levels were calculated by integrating read counts on the exons of RefSeq defined genes using HOMER. Cluster 3.0 and Java TreeView were used to cluster the gene expression values with a false discovery rate (FDR) of less than 0.05. Pathway analysis was performed by QIAGEN’s Ingenuity Pathway Analysis (IPA, QIAGEN, Redwood City, CA, USA, www.qiagen.com/ingenuity) software. Pathway analyses were performed on coding genes with two or more-fold change in expression and an FDR less than 0.05. RNA-Seq data are deposited at the National Center for Biotechnology Information (NCBI) Gene Expression Omnibus (GEO) repository, with accession number GSE155045.

### 4.7. Mass Spectrometry

NOD and B6 ES cells were collected using Trypsin EDTA and cell lysates were prepared in radioimmunoprecipitation assay (RIPA) buffer (150 mM NaCl, 50 mM Tris pH 7.5, 1% Triton X-100, 0.5% sodium deoxycholate and 0.1% SDS). Protein concentrations of supernatants were determined by BCA (Thermo Fisher). For each sample, 100 μg of lysate was washed by chloroform/methanol precipitation. Air-dried pellets were resuspended in 1% RapiGest SF (Waters) and brought up in 100 mM HEPES (pH 8.0). Proteins were reduced with 5 mM Tris(2-carboxyethyl)phosphine hydrochloride for 30 min and alkylated with 10 mM iodoacetamide for 30 min at ambient temperature and protected from light. Proteins were digested for 18 hr at 37 °C with 2 μg trypsin (Promega, Madison, WI, USA). After digestion, 20 μg of peptides from each sample was reacted for 1 hr with the appropriate TMT-6plex NHS isobaric reagent (Thermo Fisher) in 40% (*v*/*v*) anhydrous acetonitrile and quenched with 0.4% NH_4_HCO_3_ (*w*/*v*) for 1 hr. Samples with different TMT labels were pooled and acidified with 5% formic acid. Acetonitrile was evaporated on a SpeedVac and debris was removed by centrifugation for 30 min at 20,000× *g*. MudPIT microcolumns were prepared and samples were loaded onto the column offline using a pressure chamber [53]. LCMS/MS analysis was performed using a Q Exactive mass spectrometer equipped with an EASY nLC 1000 (Thermo Fisher). MuDPIT experiments were performed by 5 min sequential injections of 0, 10, 20, 30, 40, 50, 60, 70, 80, 90, 100% buffer C (500 mM ammonium acetate in buffer A) onto the MudPIT column. Each step was followed by a gradient from buffer A (95% water, 5% acetonitrile, 0.1% formic acid, *v*/*v*/*v*) to buffer B (20% water, 80% acetonitrile, 0.1% fomic acid, *v*/*v*/*v*). Electrospray was performed directly from the analytical column by applying a voltage of 2.5 kV with an inlet capillary temperature of 250 °C and S-lens RF level 50. Data-dependent acquisition of MS/MS spectra was performed with the following settings: eluted peptides were scanned from 400 to 1800 *m*/*z* with a resolution of 70,000; AGC target 1e5, max. IT 10ms and the mass spectrometer in a data-dependent acquisition mode. The top ten peaks for each full scan were fragmented by HCD using a normalized collision energy of 30%, isolation window of 2.0 *m*/*z*, a resolution of 17,500, AGC target 1e5 and max. IT 120ms, and scanned from 100 to 1800 *m*/*z*. Dynamic exclusion duration was set to 60 s. Peptide identification and TMT-based protein quantification was performed using the Integrated Proteomics Pipeline Suite IP2 (Integrated Proteomics Applications, Inc., San Diego, CA, USA) and modules ProLuCID, DTASelect and Census [54]. MS2 spectra were extracted from Thermo XCalibur.raw file format using RawConverter [55]. Spectra were searched using ProLuCID against a Uniprot mouse proteome database. The database was curated to remove redundant protein and splice-isoforms. Searches were carried out using a decoy database of reversed peptide sequences and the following search parameters: 50 ppm peptide precursor tolerance, 0.6 Da fragment mass tolerance, 6 amino acid minimum peptide length, trypsin cleavage (max. 2 missed cleavage events), static Cys modification of 57.0215 (carbamidomethylation) and static N-terminal and Lys modification of 229.1629 (TMT-6plex). ProLuCID search results were filtered using DTASelect using combined XCorr and DeltaCN scores to minimize the peptide false discovery rate at 1% and a minimum of 2 peptides per protein ID. TMT reporter ion intensities were extracted in Census using a mass tolerance of 0.05 Da, normalized across TMT channels based on total peptide abundance and then summed for individual peptides belonging to the same protein. Protein abundances were log-transformed and scaled to a common pooled reference channel that was included in each mass spectrometry sample. Expression changes were calculated as the difference between mean log2 abundances of B6 and NOD samples. Significant expression changes were accessed by unpaired student t-test followed by Benjamini–Hochberg multiple testing correction. The mass spectrometry proteomics data have been deposited to the ProteomeXchange Consorium via the PRIDE [56] partner repository with the dataset identifier PXD020055.

### 4.8. Low Input RNA-Seq and Analysis

SMART-Seq v4 Ultra Low Input RNA kit for Sequencing (Takara Bio, Kusatsu, Japan) was used to prepare low-input RNA-Seq libraries in pre-implantation embryos according to the manufacturer’s instructions. Single ICMs were stored in lysis buffer (10% Triton X-100 in water, with RNasin Plus RNase inhibitor (5% *v*/*v*)), flash-frozen and stored in -80C until library preparation. Reverse-transcribed cDNA was amplified with 19 cycles of PCR, quantified by Agilent Tapestation and then processed using Nextera XT DNA Library Preparation Kit (Illumina, San Diego, CA, USA). Libraries were sequenced on HiSeq 2500 (Illumina). Raw data were aligned to the mouse reference genome (mm10) using STAR (v2.4.2a) [57]. Differential gene expression analysis was performed using the HOMER software pipeline (v4.8) [58]. Functional protein association networks analysis was performed using STRING [59]. Gene enrichment analysis was performed using Database for Annotation, Visualization, and Integrated Discovery (DAVID) Bioinformatics Resources (v6.7) [60]. Low-input RNA-Seq data are deposited at the National Center for Biotechnology Information (NCBI) Gene Expression Omnibus (GEO) repository, with accession number GSE155044.

### 4.9. Chromatin Immunoprecipitation Coupled with Sequencing (ChIP-Seq)

ChIP-Seq experiments on ES cells were carried out similarly as described previously [34,61]. Briefly, ES cells were cross-linked with final 1% formaldehyde, then quenched and washed three times to remove residual formaldehyde. ES cell pellets were aliquoted and flash-frozen in -80C. Cross-linked ES cells were thawed and lysed in 1% SDS lysis buffer. Whole cell lysates were then sonicated on an Epishear Probe Sonicator (Active Motif, Carlsbad, CA, USA) at 10 s on / 30 s off, 40 pulses total on ice. ChIP lysates were then diluted with ChIP dilution buffer and incubated with respective antibodies and magnetic protein A beads overnight. Antibody–DNA complexes were washed with low-salt buffer, LiCl buffer and Tris buffer, then eluted, reverse-cross-linked and prepared into sequencing libraries using NEBNext Ultra II DNA Library prep kit (NEB, Ipswich, MA, USA). Antibodies used were H3K4me3 (Abcam ab8580, lot GR240214-2) and H3K27ac (Active Motif 39133, lot 31814008). ChIP-Seq data were mapped to the mouse reference genome mm10 using bowtie2 then further analyzed using the HOMER ChIP-Seq pipeline with default settings (v4.8) [58]. Super enhancer analysis was carried out using the HOMER super enhancer pipeline. ChIP-Seq data are deposited at the National Center for Biotechnology Information (NCBI) Gene Expression Omnibus (GEO) repository, with accession number GSE154458.

### 4.10. Luminex Measurement of Cytokines and Chemokines

Media were collected from ES cell cultures either 24 h after culture without any triggers or 48 h after stimulation with indicated triggers. Then, ES cultures were subjected to multiplexed antibody-based immunoassay using Luminex xMAP mouse panels (Biorad). The concentration of cytokines and chemokines were determined using standard curves, and measurements were performed in triplicates. LPS was administered at 10ug/mL (Sigma). P-values were calculated using unpaired *t* test.

## 5. Conclusions

We report on the derivation and characterization of ES cells from diabetes-prone NOD and diabetes-resistant B6 inbred mouse strains. Strikingly, we found γ-crystalline D and several T1D-associated genes to be overexpressed in NOD. Our findings suggest that susceptibility to both cataract and T1D are genetically and epigenetically inherited in NOD, and that stress/triggers can induce an aberrant expression of disease-relevant genes. Furthermore, great care must be taken when considering patient-derived cells for genetic predisposed diseases, such as deriving pancreatic beta-cells from patients with type-1 diabetes using either induced pluripotent stem cells (iPS) or transdifferentiation approaches, as genetic and epigenetic instability might be triggered in some patients. We hypothesize that ES cells from NOD and B6 can be used to study genetic instability, to identify stressors and triggers, as well as to identify potential molecules that might mediate genetic stability and thus provide a safer approach in using patient-derived cell therapies.

## Supplementary Materials

Supplementary materials can be found at https://www.mdpi.com/1422-0067/21/16/5743/s1. Figure S1: (a) Alkaline phosphatase expression in NOD and B6 ES cells. (b) NOD and B6 ES cells can form embryoid bodies with similar efficiency. (c) Quantitative real time PCR showed that all ES cells have high expression of pluripotent genes and lack genes that express in the three germ layers indicating differentiation. (d) Immunofluorescence shows similar protein expression level of pluripotent genes in B6 and NOD ES cells. (e) B6 ES cells were injected into albino blastocyst. Resulting chimeric mice were crossed with albino mice. Non-albino offspring (arrow) indicated transmission of B6 ES cells. B NOD ES cells were injected in non-albino blastocysts. Resulting chimeric mice were bred with albino mice. Albino offspring (white circles) indicated germline transmission of NOD ES cells.; Figure S2: Heatmap of all RNA-Seq data from NOD, B6, NZB and V6.5 ES cells compared to RNA-Seq data from various mouse cells and tissues obtained from ENCODE consortium. Figure S3: Hi-C data in mouse ES cells showed TADs identified in (a) Idd1, (b) Idd4.2Q, (c) Idd9.1 and (d) Idd16.1 regions. Figure S4: UCSC genome browser tracks show comparable H3K4me3 ChIP-Seq signals in up-regulated genes with CpG island at their promoters in NOD compared to B6 ES cells. Figure S5: Multiplex ELISA measurements of cytokines and chemokines which were secreted by B6 or NOD ES cells (a) at steady state, or (b) in the presence of various triggers, but show no significance difference upon stimulation by various triggers.

## Abbreviations

NOD	Non-obese diabetic mice
NZB	New Zealand black mice
RNA-Seq	RNA Sequencing
