# Supplementary material for: Premature Activation of Immune Transcription Programs in Autoimmune-Predisposed Mouse Embryonic Stem Cells and Blastocysts
Source: Int J Mol Sci. 2020 Aug 11;21(16):5743. doi: 10.3390/ijms21165743 (PMC7460978; doi:10.3390/ijms21165743)
Supplement: Supplementary file 1 [file ijms-21-05743-s001.pdf]

Supplementary Data

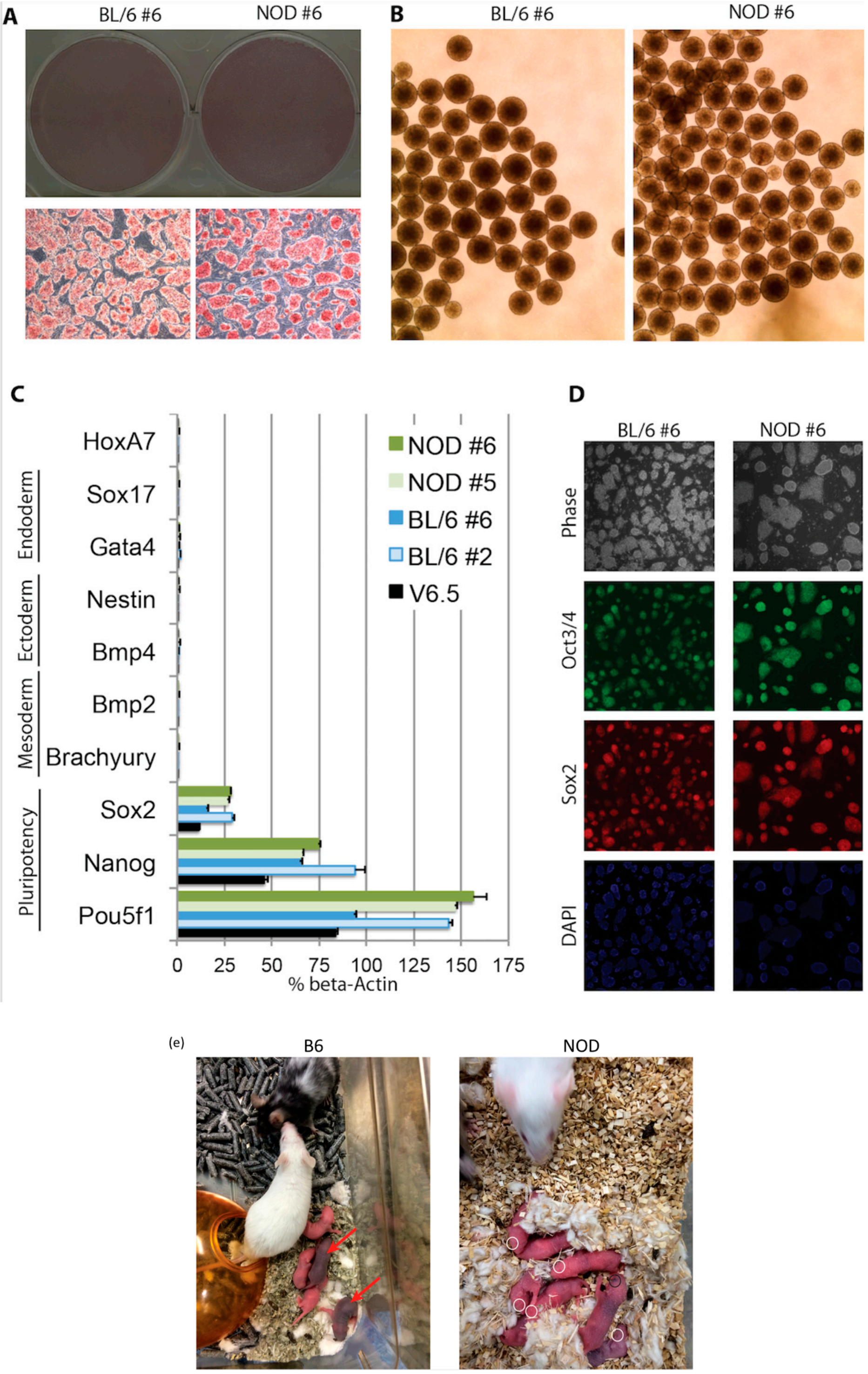

Figure S1 (a) Alkaline phosphatase expression in NOD and B6 ES cells. **(b)** NOD and B6 ES cells can form embryoid bodies with similar efficiency. **(c)** Quantitative real time PCR showed that all ES cells have high expression of pluripotent genes and lack genes that express in the three germ layers indicating differentiation. **(d)** Immunofluorescence shows similar protein expression level of pluripotent genes in B6 and NOD ES cells. **(e)** B6 ES cells were injected into albino blastocyst. Resulting chimeric mice were crossed with albino mice. Non-albino offspring (arrow) indicated transmission of B6 ES cells. B NOD ES cells were injected in non-albino blastocysts. Resulting chimeric

mice were bred with albino mice. Albino offspring (white circles) indicated germline transmission of NOD ES cells.

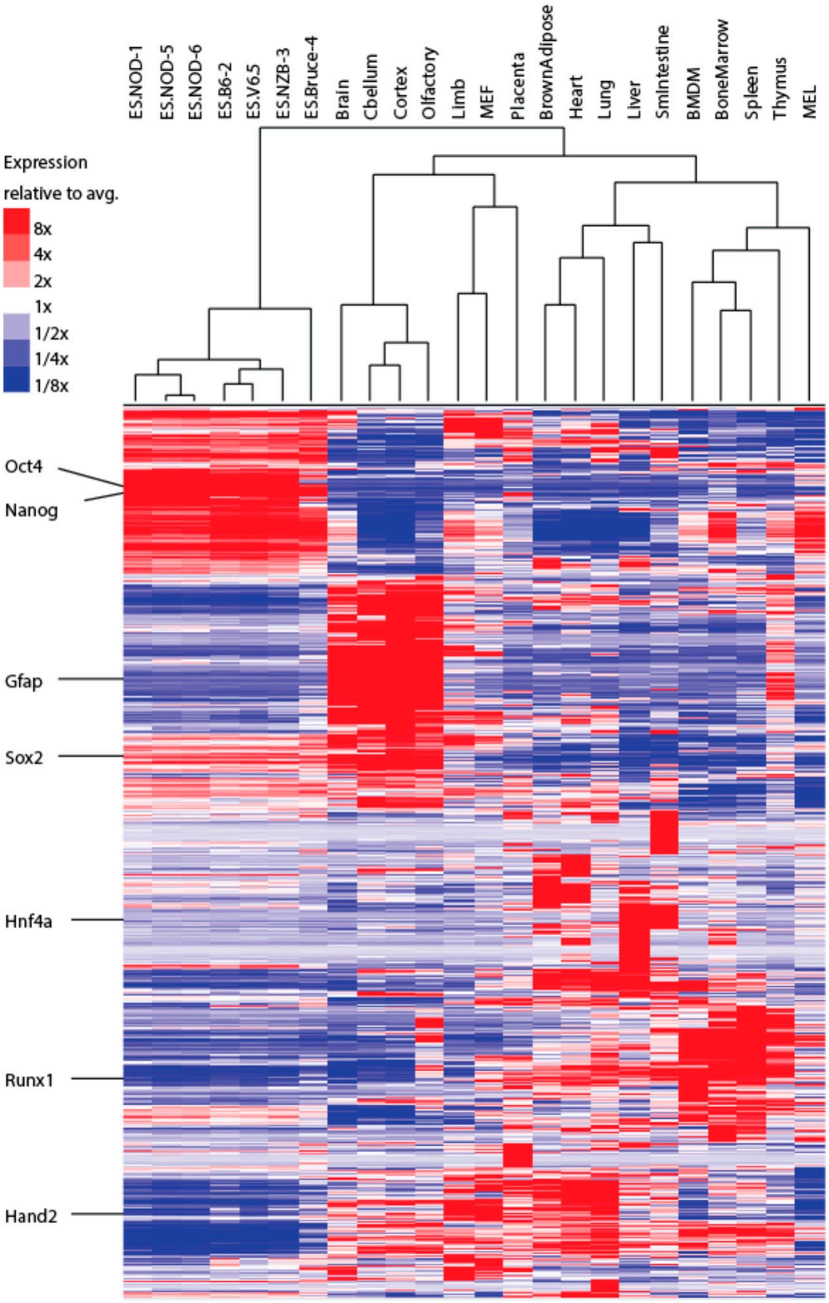

Figure S2 Heatmap of all RNA-Seq data from NOD, B6, NZB and V6.5 ES cells compared to RNA-Seq data from various mouse cells and tissues obtained from ENCODE consortium.

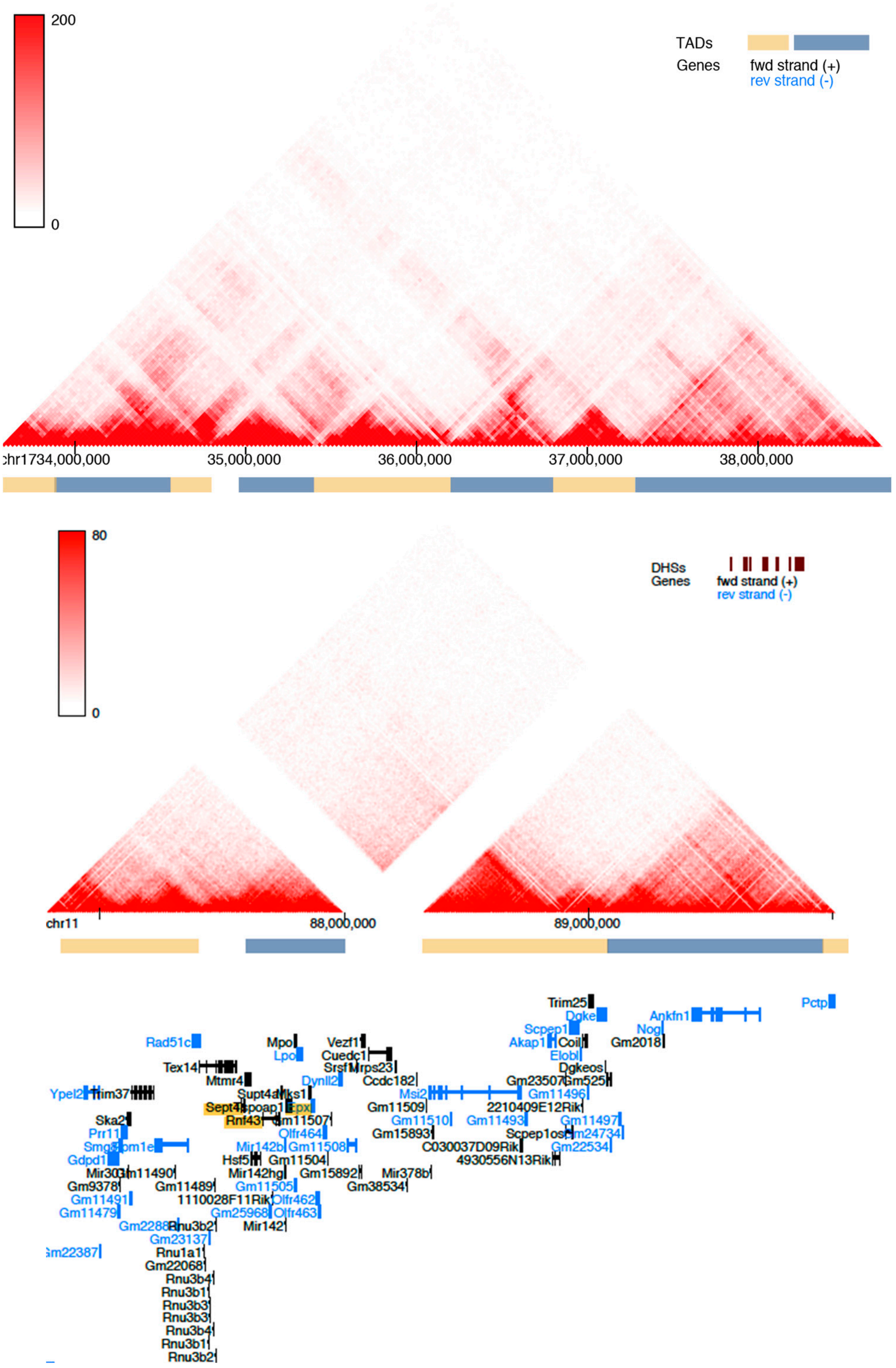
200
0
TADs
Genes
fwd strand (+)
rev strand (-)
34,000,000
35,000,000
36,000,000
37,000,000
38,000,000
80
0
DHSs
Genes
fwd strand (+)
rev strand (-)
chr11
88,000,000
89,000,000
Trim25
Pctp
Ankfn1
Nog
Gm2018
Rad51c
Mpo
Vezf1
Lpo
Cuedc1
Tex14
Mrps23
Mtmr4
Dynll2
Ccdc182
Msi2
Gm11509
2210409E12Rik
Gm11496
Gm11510
Gm11493
Gm11497
Gm15893
C030037D09Rik
Gm22534
4930556N13Rik
Dgkeos
Elob
Coil
Akap1
Scpep1
Ypel2
Ska2
Prr11
Gdpd1
Mir142b
Gm11508
Hsf5
Gm11504
Mir142hg
Gm15892
Mir378b
Gm9378
Gm11489
Gm11505
Gm38534
Gm11491
1110028F11Rik
Olfr462
Gm11479
Gm25968
Olfr463
Mir142
Gm23137
Rnu1a1
Gm22068
Rnu3b4
Rnu3b1
Rnu3b3
Rnu3b2
Olfr464
Rnf43
Sept4

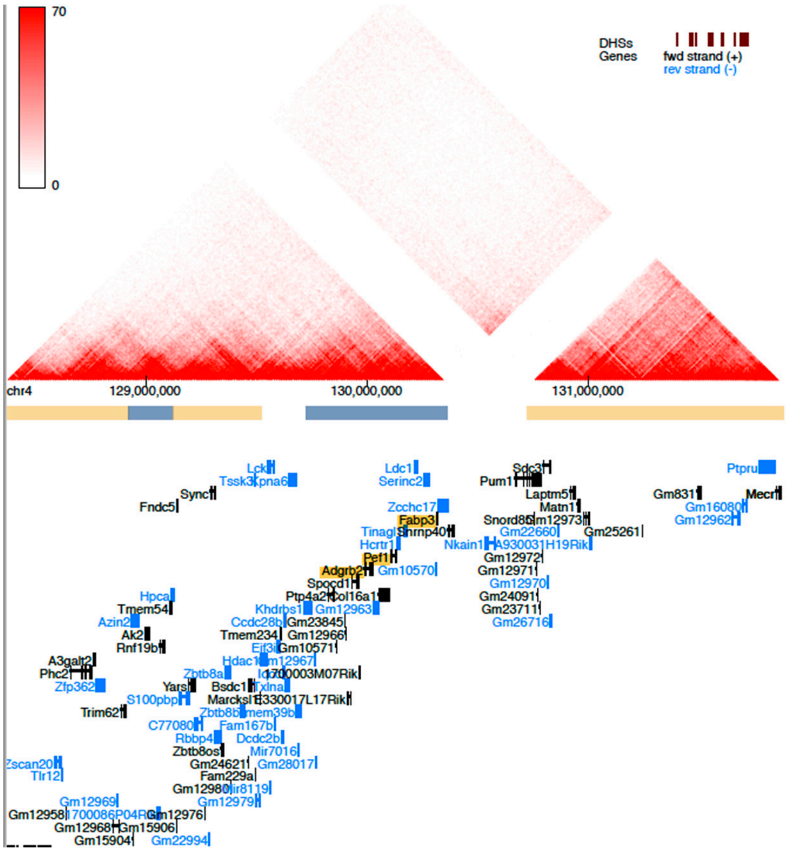

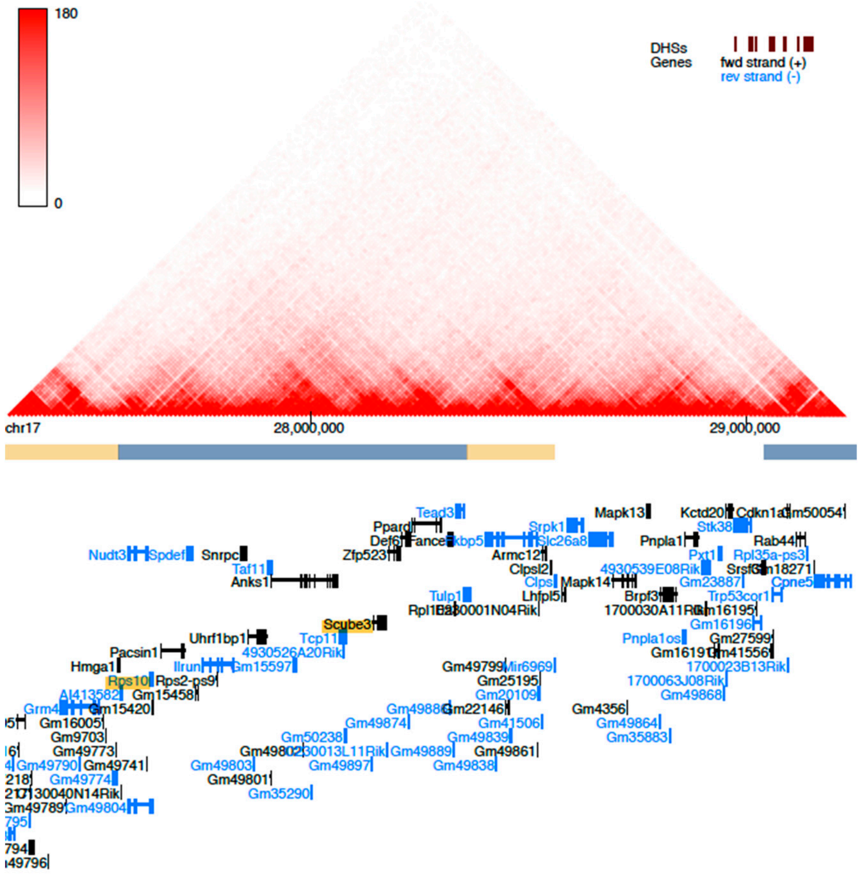

Figure S3 Hi-C data in mouse ES cells showed TADs identified in (**a**)Idd1, (**b**)Idd4.2Q, (**c**)Idd9.1 and (**d**)Idd16.1 regions.

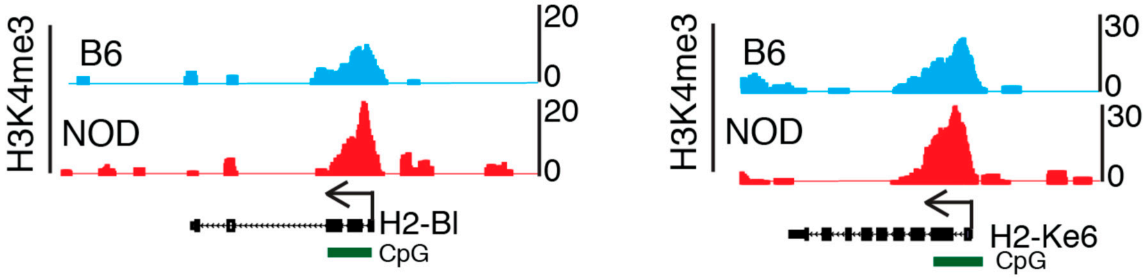

Figure S4 UCSC genome browser tracks show comparable H3K4me3 ChIP-Seq signals in up-regulated genes with CpG island at their promoters in NOD compared to B6 ES cells.

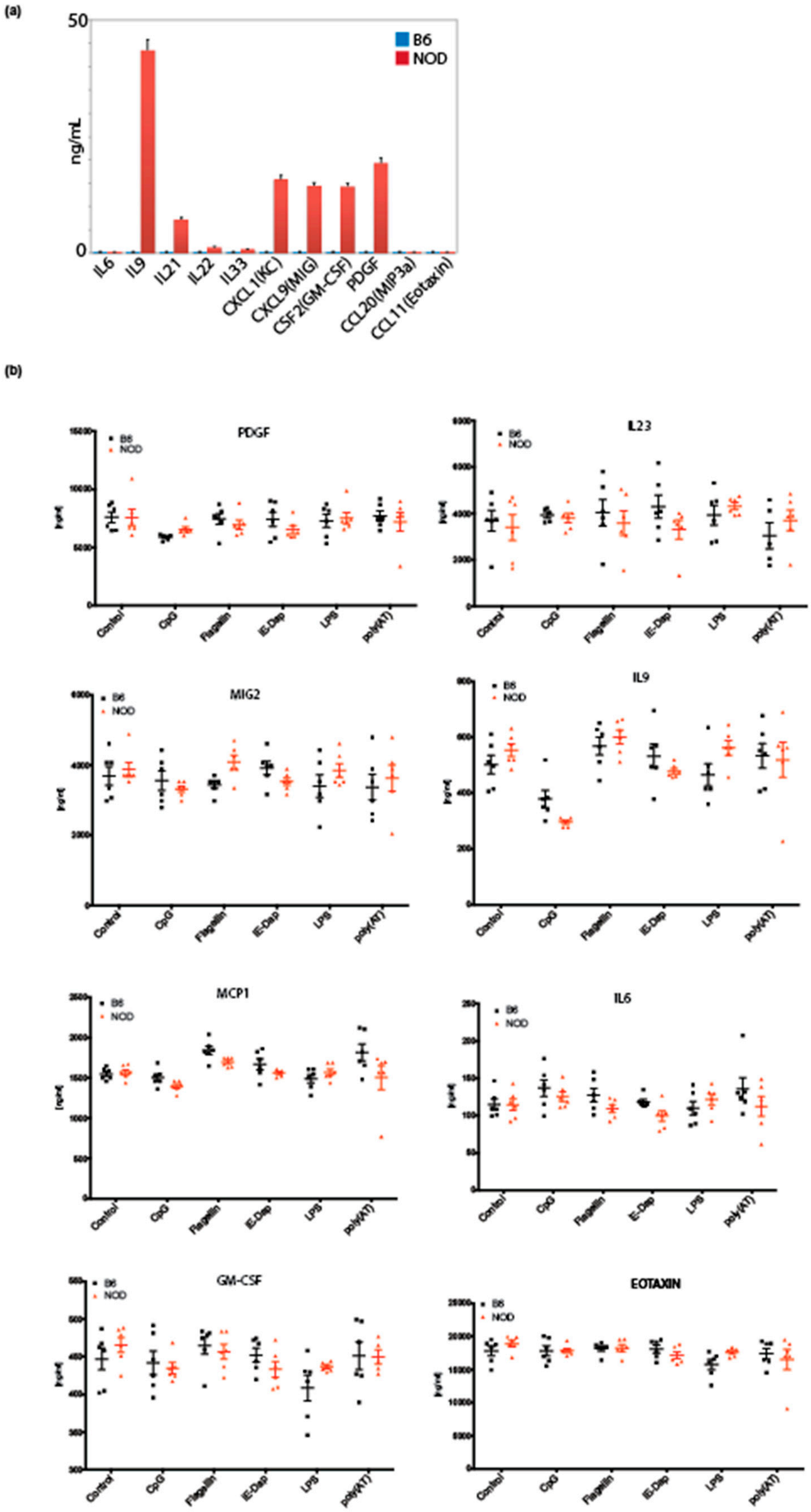

Figure S5 Multiplex ELISA measurements of cytokines and chemokines which were secreted by B6 or NOD ES cells (a) at steady state, or (b) in the presence of various triggers, but show no significance difference upon stimulation by various triggers.
